# Extent and predictors of presenteeism among healthcare professionals working in Swiss hospitals, nursing homes and home care organizations

**DOI:** 10.1038/s41598-023-39113-6

**Published:** 2023-07-25

**Authors:** Karin Anne Peter, Maisa Gerlach, Gablu Kilcher, Reto Bürgin, Sabine Hahn, Christoph Golz

**Affiliations:** 1 Department of Health Professions, Bern University of Applied Sciences, Bern, Switzerland; 2 Department Health Services Research, SWICA Health Organization, Winterthur, Switzerland; 3 Institute of Data Analysis and Process Design, Zurich University of Applied Sciences, Winterthur, Switzerland

**Keywords:** Health care, Health occupations

## Abstract

Presenteeism can have negative impacts on employees’ health and organizational productivity. It occurs more often among occupations with high attendance demands, such as healthcare professionals. Information is lacking regarding the extent to which presenteeism differs between disciplines and settings in the health sector and what the reasons are for presenteeism as well as influencing factors. This study used cross-sectional data on 15,185 healthcare professionals (nursing staff, midwives, physicians, medical-technical and medical-therapeutic professionals) from various settings (acute care, rehabilitation or psychiatric hospitals, nursing homes and home care organizations). Presenteeism was measured by examining how many days participants had gone to work despite feeling sick during the past 12 months. Kruskal–Wallis was used to test for significant differences between healthcare professions/settings and regression analysis to identify significant predictors of presenteeism. Nursing assistants with a formal education reported the most days of presenteeism in the past 12 months (mean = 4.3, SD = 12.0). Healthcare professionals working in nursing homes reported the most days of presenteeism in the past 12 months (mean = 4.2, SD = 8.7). The majority of healthcare professionals had been present at work while being ill due to a sense of duty (83.7%), followed by consideration for colleagues and/or managers (76.5%). In particular, the psychiatric hospitals (β = 0.139; p < 0.001), nursing homes (β = 0.168; p < 0.001) and home care organizations (β = 0.092; p < 0.001), as well as the language regions of Swiss French (β = − 0.304; p < 0.001) and Italian (β = − 0.154; p < 0.001), were significantly associated with presenteeism. Presenteeism differs between disciplines and settings in the health sector. The reasons for presenteeism and its influencing factors in the health sector are mostly consistent with those in other sectors. Cultural differences should be afforded greater relevance in future presenteeism research.

## Introduction

In recent years, the investigation of presenteeism has attracted increasing attention due to its negative impact on employees’ health and organizational productivity^1–3^. In contrast to presenteeism, absenteeism, which refers to not showing up for work, has been widely researched in the last few decades^3^. However, some authors claim that presenteeism leads to a far greater aggregate productivity loss than absenteeism^4–6^. In Switzerland, presenteeism accounted for approximately two thirds of the total health-related production losses in 2016, which is close to three times the cost of absenteeism (measured using the Work Productivity and Activity Impairment Scale)^7^.

Until now, there has not been a consensus over a definition or a consistent measurement method established in research^3^. Currently, there are two dominant perspectives on presenteeism. The North American perspective sees presenteeism as a productivity loss due to the reduced performance of workers with untreated health problems. This approach is often used to monetize the costs of presenteeism. However, the appropriateness of measuring health-related productivity losses and calculating their costs is subject to criticism^8^. In European research, presenteeism is predominantly understood as the behavior of going to work despite illness^8,9^. In addition to those two dominant perspectives, a third perspective emerges that sees presenteeism as multidimensional^10,11^. This definition widens the understanding of presenteeism to being not solely illness related or associated with reduced performance^8,10^. In this study, we refer to the European line of research with the understanding of presenteeism as a behavior of going to work despite illness and not as the “impact of the individuals’ health condition on their productivity and the financial loss for the organization” [^8^, p. 346].

A European study about working conditions found that 40% of the respondents had worked while they were sick for at least one day in the previous 12 months, with women stating that they go to work more often while being sick^12^. Presenteeism was found to occur more often among occupations with high attendance demands or the so-called “helping professions,” such as healthcare professionals^13^. For example, Chambers, Frampton and Barclay^14^ identified a prevalence of 88% for presenteeism among healthcare professionals. The higher prevalence among healthcare professionals might be accentuated by the fact that women make up the majority of healthcare professionals and are prone to presenteeism^15^.

The influencing factors of presenteeism^3,8^, such as quantitative or emotional demands and the work-privacy conflict, correspond to those identified among health professionals^16^. The consequences of presenteeism include reduced mental and physical health among health professionals and decreased patient safety^17^. However, a recent literature review found that the number of available studies on presenteeism among healthcare professions, its influencing factors and reasons is low and the majority focus only on nurses, thereby neglecting other disciplines^18^. Furthermore, the level of presenteeism seems to differ between settings, as it was found that healthcare professionals working in hospital settings reported a higher rate of presenteeism than those in long-term care^19^. With regard to the aforementioned influencing factors of presenteeism, healthcare professionals are affected by many of these, including high emotional and physical demands, working under time pressure, long working hours, work-private life conflicts, aggressive patients and visitors, and exposure to infectious diseases and/or hazardous substances^20–22^. However, the work-related stress experienced by health professionals differs between disciplines and work areas^23^. This leads to the question of whether presenteeism differs between disciplines and work areas in the health sector and to identifying relevant predictors of presenteeism among healthcare professionals working in hospitals, nursing homes and home care organizations.

The aim of this study, therefore, was to identify: (1) the extent of presenteeism among different healthcare professional work areas and disciplines: (2) the reasons for presenteeism; and (3) predictors of presenteeism among Swiss healthcare professionals working in Swiss acute care, rehabilitation or psychiatric hospitals, nursing homes and home care organizations.

## Method

### Design

This study is based on a cross-sectional study design and is part of the national STRAIN study—work-related **STR**ess **A**mong health professionals **IN** Switzerland (Clinical Trials registration: NCT03508596, cluster RCT). The STRAIN study consists of three data measurements (T^0^, T^1^, T^2^) and data were collected from September 2017 to March 2018 (T^0^), from January to April 2019 (T^1^) and from March to September 2020 (T^2^). Participating organizations were free to choose the time that suited them best during the data collection period T^0^–T^2^. For this study, all STRAIN measurements (T^0^, T^1^, T^2^) were included^23,24^. The proportion of repeated participation across the three measurements was low, as only 4% of the participants took part in all measurement periods. Thus, we merged the measurement periods into one data set. We adhered to the STROBE (STrengthening the Reporting of OBservational studies in Epidemiology) checklist (see Supplementary Information).

### Recruitment of healthcare organizations

This study consists of the same sample as the STRAIN study. For recruitment, all registered hospitals, nursing homes and home care organizations were selected from a list provided by the Swiss Federal Statistical Office in 2016. Organizations that were too small (average number of beds < 20, fewer than 7 employees), or that were specialized (e.g., in gynecology or neonatology), were excluded. Computer-based randomization (randomizer.org) was conducted, and a total of 100 hospitals (acute, rehabilitation and psychiatric), 100 nursing homes and 100 home care organizations were invited to participate. Consideration was also given to ensuring a geographically representative sample of Switzerland (69% Swiss or standard German-speaking, 23% French-speaking, 8% Italian-speaking)^23^.

The selected healthcare organizations received information about the study by email or telephone. Afterwards, a flyer and a short film containing information about the study were sent directly to the CEO or the head of human resources. A total of 26 acute care/rehabilitation and 12 psychiatric hospitals (23 German-speaking, 12 French-speaking, 1 Italian-speaking) took part in this study. Additionally, 86 nursing homes (56 German-speaking, 24 French-speaking, 6 Italian-speaking) and 41 home care organizations (36 German-speaking, 3 French-speaking, 2 Italian-speaking) participated^24^.

### Study sample and data collection

For data collection, a contact person in each participating organization was responsible for distributing the questionnaires, which were sent to all nursing staff, midwives, physicians, medical-technical and medical-therapeutic professionals at all skill levels. A short film and a written study flyer were used to inform them about the study. The questionnaire was available in German, French and Italian, using an online version and a printed paper version with a direct reply envelope. Participants had one month to complete the questionnaire and they received a reminder after two weeks had passed.

### Questionnaire

For this study the STRAIN questionnaire was used, which is based on the theoretical framework “causes and consequences of work-related stress” from Eurofound^25^ and consists of well-established, valid and reliable scales from the Copenhagen Psychosocial Questionnaire—COPSOQ^26–28^, the questionnaire from the Nurses Early eXit sTudy (NEXT)^29^, the Oslo Social Support Scale (Oslo-3)^30,31^ and the Sixth European Working Conditions Survey (EWCS)^32^. To measure presenteeism as a behavior, researchers predominantly refer to single items^8^. In particular, the single item defined by Aronsson, Gustafsson and Dallner^13^ of understanding the behavior as dysfunctional has been used the most^33^. The item “How many days have you gone to work despite feeling that you really should have taken sick leave due to state of health?” has been used with various response formats and recall periods^8^. In this study we use the item used in the NEXT questionnaire^29^ to allow comparisons to be made between the results of two German-speaking neighboring countries. It is based on the single item described above^13^ and has a recall period of 12 months and a response format in days raging between 0 and 365 days): “In the last 12 months, how many days have you gone to work despite feeling that you really should have taken sick leave due to your state of health?” Furthermore, we used the response format in numbers of days from 0 to 365, since other known formats have been criticized before being too crude, which makes it difficult to measure presenteeism adequately, since it is known to have low values in reporting^34^. Although single items measuring presenteeism often lack proper psychometric evaluation, particularly in terms of validity^8^, a meta-analysis aimed at establishing the reliability of such scales reported an acceptable reliability of 0.79^1^.

To identify the most important reasons why healthcare professionals decided on presenteeism, we developed in-house items (according to the latest results from the Swiss State Secretariat for Economic Affairs (SECO): “For what reasons did you go to work anyway?” (multiple answers possible): (1) Sense of duty; (2) Because otherwise work would be left undone; (3) Consideration for colleagues and/or managers; (4) Fear of professional disadvantages; (5) Fear of job loss; (5) Other reasons (free text box). These items only serve as possible answer choices for the participants and do not result in a scale.

### Analysis

Data were analyzed using R 3.6.0. The mean of all scales (COPSOQ and EWCS) was transformed to a value ranging from 0 (minimum value) to 100 (maximum value) points from the initial ranges of 1–5 (COPSOQ) and 1–7 (EWCS). No average score was calculated if less than half of the questions in a scale had been answered^26^. Other items were dummy coded (1 = yes, 0 = no).

First, descriptive statistics regarding the (a) study sample and the extent of presenteeism among different (b) healthcare professionals and (c) healthcare settings were computed and further tested for significant differences. Since the test of homogeneity of variance was significant and there were no equal-sized samples of data, the Kruskal–Wallis test (using Bonferroni correction for multiple tests) was used to test for significant differences.

Second, reasons for presenteeism were analyzed using descriptive statistics. For data analysis, French and Italian free texts were translated into German, verified by native speakers and summarized using the content analysis approach^35^.

Third, predictors of presenteeism were analyzed using regression analysis. The response variable of presenteeism involves count data and the test for zero inflation was significant (p < 0.001), indicating that more zeros were found in the data set than expected^36^. Thus, we compared the following three models: zero-inflated Poisson, Poisson and linear regression with log transformation. The comparison of the three models revealed that the linear model yielded very similar regression coefficients to both the Poisson model and the zero-inflated Poisson model. The fact that zero-inflated models reveal only small differences compared to others has been discussed and argued in favor of less complex models to facilitate the interpretation^36^. Thus, a linear model with logarithm transformation was used for the next steps.

**Figure 1:**
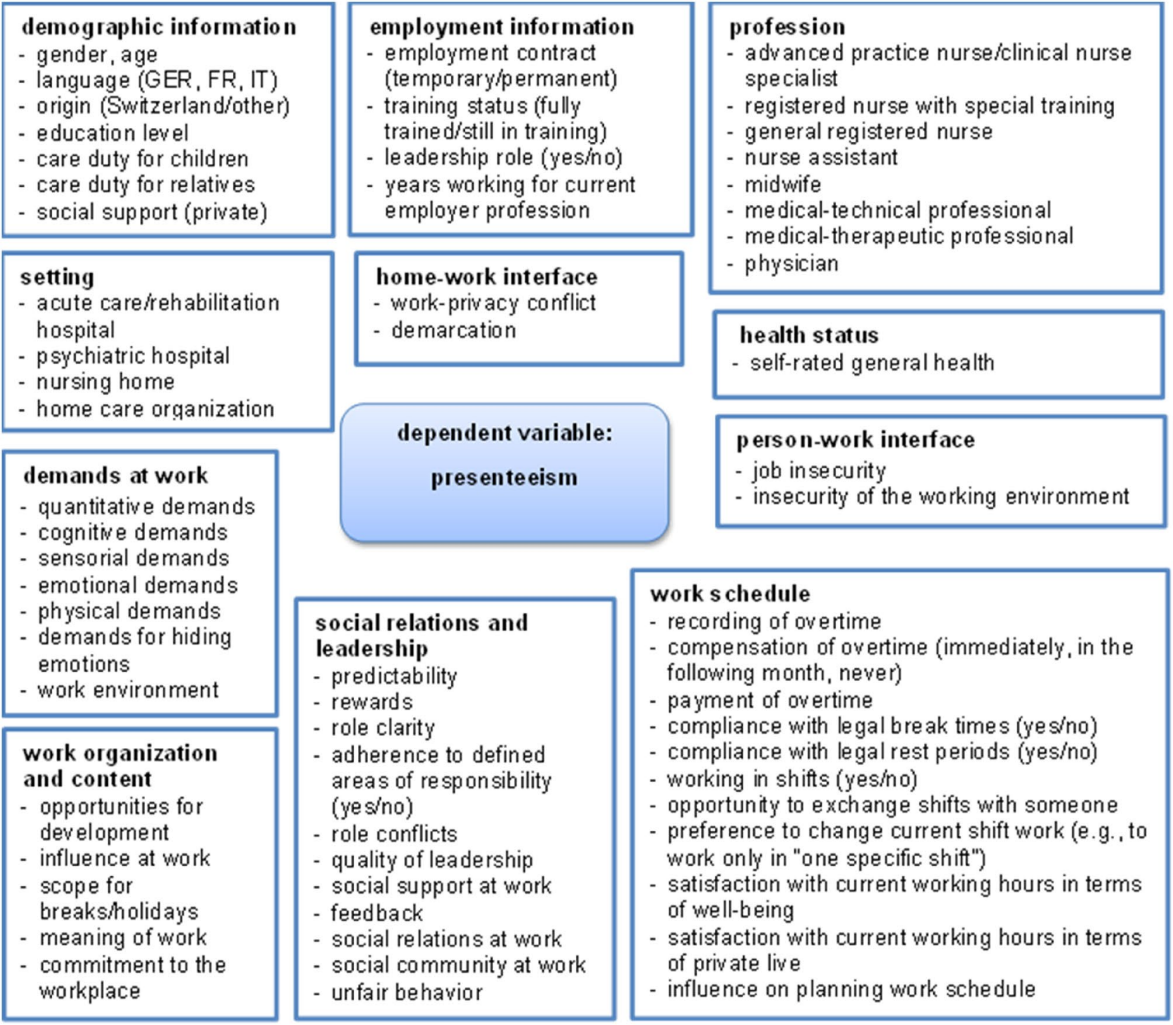
Possible predictors in the regression analysis.

The initial variable selection of the predictors was based on the framework model of presenteeism by Lohaus and Habermann^3^, resulting in a set of 66 potential explanatory variables. Thus, several scales and single items on “demographic” and “employment” information, healthcare professionals’ “work schedule” and “clinical settings” as well as various “demands at work,” “social relations and leadership,” “person-work interface factors” and “work organization and content” were used as potential predictors of presenteeism (Fig. 1). We focused on a two-level hierarchical model since the data have a hierarchical structure where healthcare professionals are nested within health services. Although the health services could have been defined as being nested in the setting, we used the setting as a fixed effect, since the minimum needed number of groups per level should be five and we only have four in the setting^37^. For further variable selection, we performed backward selection by minimizing the Bayesian Information Criterion (BIC) criteria^38^. BIC criteria were used since they are known to be more restrictive than AIC^39^ and we wanted to have a selection from the initial theory-based variable pool for a simpler model. The BIC chooses the threshold according to the effective sample size. If removing a variable would result in a decrease in the BIC, it is excluded to reach a balance between goodness of fit and model complexity. Missings were excluded listwise.

The final regression model was analyzed utilizing confidence intervals of the estimated coefficients using the bootstrap procedure. The significance of the variables was determined by likelihood ratio tests using the bootstrap method to compute the p values. To obtain the variance explained by the regression model, we considered the marginal and conditional coefficient of determination^40^.

### Ethics approval and consent to participate

The president, Prof. Dr. med. Christian Seiler of the local Swiss ethical board in Bern, confirmed that the study does not warrant a full ethical application and does not fall under the Swiss Federal Act on Research Involving Human Beings (Req-2016-00616). The study was conducted in accordance with the Declaration of Helsinki. It was performed on a voluntary basis for all organizations and healthcare professionals participating; all participants were free to stop filling out the questionnaire at any time. Participants received written information before the start of the study about the contents, aim and voluntary nature of their participation and gave their informed consent by completing the first survey page.

## Results

### Study sample

The study sample consisted of 15,185 healthcare professionals (unique cases only) from 169 health organizations, with 83% from the German-, 15% from the French- and 2% from the Italian-speaking part of Switzerland. Most participants were female (81%) with a mean age of 40.68 years (SD = 12.70); they had an average of 17.79 (SD = 11.58) years of professional experience and 7.72 (SD = 7.93) years working in their current position. The majority (68%) of the participating healthcare professionals originated from Switzerland or from Germany (11%).

Nurses made up 71% of the study sample, with 48% being general registered nurses, 23% being nursing assistants and 7% having had no formal nursing education. Midwives made up 1% of the study sample, physicians 7%, medical-technical professionals 3%, medical-therapeutic professionals 8%, and employees from administration and research 2%. A total of 43% of the participating healthcare professionals worked in an acute care or rehabilitation hospital, 23% in a psychiatric hospital, 20% in a nursing home and 14% in a home care organization (Table 1).

**Table 1:** Sample characteristics.

	All Settings		Acute care/Rehabilitation		Psychiatric hospital		Nursing home		Home care organization	
Participants	N = 15,185		N = 6486		N = 3526		N = 3090		N = 2083	
Characteristics										
	Mean (SD)	N (%)	Mean (SD)	N (%)	Mean (SD)	N (%)	Mean (SD)	N (%)	Mean (SD)	N (%)
Age	40.68 (12.70)		38.96 (12.04)		41.13 (12.53)		41.65 (13.82)		43.84 (12.63)	
Sex										
Female		12,274 (81)	5253 (81)		2453 (70)		2638 (85)		1930 (93)	
Male		2639 (17)	1126 (17)		986 (28)		410 (13)		117 (6)	
NA		272 (2)	107 (2)		87 (2)		42 (2)		36 (1)	
Professional experience	17.79 (11.58)		18.36 (11.34)		17.35 (11.48)		15.69 (12.11)		19.56 (11.34)	
Current position (years)	7.72 (7.93)		8.1 (8.37)		6.81 (7.14)		7.89 (8.02)		7.83 (7.54)	
Profession										
Nurses		10,781 (71)		4021 (62)		1949 (55)		2827 (91)		1984 (95)
Midwives		152 (1)		152 (2)		0 (0)		0 (0)		0 (0)
Physicians		1063 (7)		617 (10)		409 (12)		37 (2)		0 (0)
Medical-technical		456 (3)		456 (7)		0 (0)		0 (0)		0 (0)
Medical-therapeutic		1215 (8)		536 (8)		561 (16)		118 (4)		0 (0)
Administration & research		304 (2)		190 (3)		64 (2)		12 (< 1)		38 (2)
NA		1214 (8)		514 (8)		543 (15)		96 (3)		61 (3)
Language region										
German-speaking		12,616 (83)		5141 (79)		3283 (93)		2467 (80)		1725 (83)
French-speaking		2286 (15)		1345 (21)		86 (2)		540 (17)		315 (15)
Italian-speaking		283 (2)		0 (0)		157 (5)		83 (3)		43 (2)

### Extent of presenteeism among different healthcare professions and settings

Results regarding significant differences between registered nurses, nursing assistants, midwives, physicians, medical-technical professionals, medical-therapeutic professionals, and employees from administration and research using the Kruskal–Wallis test are presented in Table 2. The results showed the highest mean values for nursing assistants with a formal education and the lowest among midwives, medical-technical professionals and medical-therapeutic professionals (see also pairwise comparison in Table 2 for significant differences).

**Table 2:** Extent of presenteeism among different professions and settings.

Presenteesism (number of days 0–365)	N	Mean	SD	Median	Kruskal–Wallis test	
Professions					p value	Significant differences using pairwise comparison*
Registered nurses^1^	5922	3.4	6.6	2	< 0.001	1vs2; 1vs7; 2vs3; 2vs5; 2vs6; 2vs7
Nurse assistants with formal education^2^	3030	4.3	12.0	2		
Nurse assistants without formal education^3^	1018	3.1	5.3	2		
Midwives^4^	133	2.7	3.6	2		
Physicians^5^	918	3.4	6.0	2		
Medical-technical professionals^6^	408	2.8	5.2	2		
Medical-therapeutic professionals^7^	1144	2.9	11.9	2		
Employees from administration/research^8^	358	3.7	6.8	2		
Settings						
Acute care/rehabilitation hospitals^1^	6486	3.3	9.8	2	< 0.001	1vs2; 1vs3; 1vs4; 2vs3; 2vs4; 3vs4
Psychiatric hospitals^2^	3526	3.5	5.6	2		
Nursing homes^3^	3090	4.2	8.7	2		
Home care organizations^4^	2083	2.7	7.0	2		

N = number of cases in total, SD = standard deviation, *pairwise comparison using the significance level of 0.05 (2-sided), adjusted by Bonferroni correction for multiple tests.

The superscript numbers 1 - 8 refer to the right column "Significant differences using pairwise comparison".

Further results regarding significant differences between acute care/rehabilitation hospitals, psychiatric hospitals, nursing homes and home care are also presented in Table 2. Significant differences using pairwise comparison were found between all healthcare settings. This revealed the highest mean values for presenteeism among healthcare professionals working in nursing homes and the lowest among healthcare professionals working in home care organizations.

### Results on reasons for presenteeism

Overall, 9533 healthcare professionals completed the items on possible reasons for presenteeism (see Table 3). The majority of participants (83.7%) named their own sense of duty as the most frequent reason for presenteeism. Another 76.5% of the participants stated that they went to work despite their illness out of consideration for colleagues and/or superiors. Around 24.4% of the healthcare professionals stated that they went to work “because otherwise the work would be left undone” as a reason for presenteeism. Fear of professional disadvantages (8.2%) and fear of losing one’s job (5.7%) were also reasons for presenteeism among healthcare professionals.

**Table 3:** Reasons for presenteeism.

Reasons for presenteeism		Number of responses (%)
Given answer selection of reasons	Sense of duty	7975 (83.7%)
	Consideration for colleagues and/or managers	7296 (76.5%)
	Because otherwise the work would be left undone	2326 (24.4%)
	Other reasons	808 (8.5%)
	Fear of professional disadvantages	783 (8.2%)
	Fear of job loss	543 (5.7%)
Other reasons	Work ethic and social pressure from the team (e.g., negative comments from the team when calling in sick)	164 (25.8%)
	Lack of staff (e.g., no replacement available)	160 (25.2%)
	Sense of commitment to patients (e.g., for the benefit of patients)	109 (17.2%)
	One does not feel sick enough (e.g., health condition is assessed as still good enough to work)	58 (9.1%)
	No replacement possible due to professional expertise (e.g., only nephrologist on duty in the hospital)	46 (7.3%)
	Expectations and pressure from the superior (e.g., being asked by the superior to show up at work despite illness)	27 (4.3%)
	Financial disadvantages (e.g., for hourly paid employees)	26 (4.1%)
	Absence management of the employer (e.g., because of the obligation to submit a medical certificate after one day of absence)	19 (3.0%)
	Distraction from being sick (e.g., because you feel sicker at home than at work)	18 (2.8%)
	Alternative work was offered (alternative work could be taken on, e.g., in the back office instead of direct patient contact)	8 (1.3%)

A total of 808 healthcare professionals chose the answer selection “other” and had the opportunity to add their own reasons to the existing answer selection. Of those, a total of 635 of the completed texts could be included in the content analysis. Those texts were written in German (83.4%), French (15.6%) and Italian (1%), mainly by nurses (57%), medical-therapeutic professionals (11.2%), medical-technical professionals (4.2%), administration and research personnel (3.5%) and midwives (1.7%). From the free text analysis, 10 other common reasons for presenteeism were identified, with work ethic and social pressure from the team (25.8%) as the other major reason, followed by a sense of commitment to patients (17.2%).

### Results of the multiple regression model on presenteeism

Results from the final hierarchical model on presenteeism are presented in Table 4 (predictors explained 25% of the variance). For demands at work, we found higher quantitative (β = 0.004, p < 0.001) and emotional (β = 0.004, p < 0.001) demands at work, as well as higher demands of having to hide feelings at work (β = 0.003 p < 0.001), which are associated with higher presenteeism. Also, a perceived stressful work environment (e.g., noise, cold) was associated with a higher level of presenteeism in everyday life (β = 0.003, p < 0.001).

**Table 4:** Results of the hierarchical model with presenteeism as the outcome variable.

Predictors		Estimate (%Δ)	Estimate (log)	SE	t value	p value*	CI (2.5%)*	CI (97.5%)*	VIF	R^2^
(Intercept)			0.941	0.093	10.174	< 0.001	0.760	1.123		0.25
Setting: psychiatry^1^		14.9	0.139	0.034	4.046	< 0.001	0.075	0.207	1.39	
Setting: nursing home^1^		18.3	0.168	0.033	5.098		0.105	0.233		
Setting: home care organization^1^		9.6	0.092	0.039	2.357		0.012	0.163		
French-speaking language region^2^		− 26.2	− 0.304	0.033	− 9.112	< 0.001	− 0.368	− 0.241	1.12	
Italian-speaking language region^2^		− 14.3	− 0.154	0.075	− 2.051		− 0.305	− 0.011		
Employment level (working hours per week)		0.3	0.003	0.000	5.989	< 0.001	0.002	0.004	1.14	
Compliance with legal break times (yes = 1)		− 10.0	− 0.105	0.024	− 4.431	< 0.001	− 0.154	− 0.061	1.07	
Profession: physician		− 13.5	− 0.145	0.040	− 3.639	< 0.001	− 0.223	− 0.067	1.10	
Profession: administration & research		20.8	0.189	0.066	2.846	< 0.007	0.052	0.319	1.02	
Quantitative demands at work		0.4	0.004	0.001	6.283	< 0.001	0.003	0.005	1.29	
Emotional demands at work		0.4	0.004	0.001	5.602	< 0.001	0.002	0.005	1.27	
Demands to hide emotions		0.3	0.003	0.000	5.860	< 0.001	0.002	0.004	1.22	
Perceived reward		− 0.20	− 0.002	0.000	− 5.167	< 0.001	− 0.003	− 0.001	1.25	
Insecurity of the working environment		0.3	0.003	0.000	6.097	< 0.001	0.002	0.004	1.34	
Work-private life conflict		0.5	0.005	0.001	9.115	< 0.001	0.004	0.006	1.65	
Demanding work environment		0.3	0.003	0.001	5.112	< 0.001	0.002	0.004	1.30	
General health status		− 1	− 0.010	0.001	− 15.822	< 0.001	− 0.011	− 0.008	1.18	
Random effects										
Std.Dev.			0.06 (0.00–0.09)							
Residuals			0.70 (0.68–0.72)							

*Based on bootstrap, ^1^settings was used as a categorial variable with acute care/rehabilitation hospitals as an indicator, ^2^language regions was used as a categorical variable with the German-speaking region as an indicator, SE = standard error, VIF = variance inflation factor, estimate (%Δ): percentage increase (or decrease) in the response for every one-unit increase in the predictor variable (exp(β1)− 1)⋅100.

In addition, the lower the perceived appreciation by superiors (β = − 0.002, p < 0.001) and the higher the uncertainty regarding working conditions (e.g., changing shift schedule) (β = 0.003, p < 0.001), the higher the level of presenteeism that was reported. With regard to work-life balance, the results show that the higher healthcare professionals rate the conflict between work and private life, the stronger their tendency towards presenteeism (β = 0.005, p < 0.001). The self-assessed health status was also shown to be a significant predictor in the regression model, whereby the worse the health status was assessed, the higher the level of presenteeism that occurred in everyday working life (β = − 0.010, p < 0.001). As regards the hypothesized need for a hierarchical approach, the estimated random effect of the organization is rather small with an estimated standard deviation of 0.06 (0.00–0.09).

When interpreting the results, it should be noted that the influence (β) of individual predictors was sometimes low. This is related to the logarithm transformation of the outcome variable as described in the analysis. For the interpretation of the regression results, we exponentiated the coefficient, subtracted one from this number and multiplied by 100. This yielded the percentage increase (or decrease) in the response for every one-unit increase in the predictor variable (see estimate (%Δ)).

## Discussion

This study presents important findings on presenteeism among Swiss healthcare professionals working in different healthcare settings, the correlations of different predictors and the reasons for presenteeism.

Internationally, predominantly data on presenteeism among nurses are available^18^. In our study the extent of presenteeism among nurses was lower than the findings from the representative nurses’ early exit study from Germany, in which 3565 nurses participated (3.6 vs. 5.03 days/year)^41^. This discrepancy might be due to the difference between the countries of Switzerland and Germany with regard to the number of nurses per 1000 population, at 11.4 vs. 10.8, respectively^42^. It has been shown that understaffing is an influencing factor of presenteeism among healthcare professionals.^43^. However, it should be noted that the results from Germany stem from 2005, which limits the interpretation due to the large time gap between the two studies. Nonetheless, no other comparable data are available, since presenteeism has been measured either with another scale^16^, response format or recall period^44^. The heterogeneity of approaches to measure presenteeism is particularly problematic for comparisons.

The results of the comparison between the disciplines show that nursing assistants with a formal education are the discipline most affected by presenteeism, closely followed by administrators and research staff, as well as nurses and doctors. This difference is contrary to other findings, concluding that registered nurses have significantly higher levels of presenteeism than nursing aides^45^. Thus, the reason for our findings could be due less to the specificity of the discipline and more to the setting in which they work, since in Switzerland the majority of nursing assistants work in long-term care^46^, and in this setting it was found that presenteeism occurred more often. A higher weighting of the setting is also evident in the regression. While the Kruskal–Wallis test shows the highest mean of presenteeism among the nurse assistants with a formal education, the nurse assistants were excluded from the final model. The higher prevalence of presenteeism in nursing homes from our findings is in line with findings from the nurses’ early exit study^41^. One reason for the setting difference might again be the higher level of understaffing in long-term care^46^. In Switzerland, while in acute care an additional 36% of nurses are needed by 2035, in nursing homes it is 49%^46^.

The most frequent reasons for presenteeism in our study overlap with the findings of Hägerbäumer^33^. In her study, Hägerbäumer^33^ surveyed 1550 employees in an acute care organization. However, it is important to note that in our study the two reasons “Fear of professional disadvantages” and “Fear of job loss” were given very little relevance. The sense of security with regard to a stable working situation could originate from the fact that the health sector is experiencing a shortage of qualified healthcare professionals and health organizations are committed to retaining these professionals^47^. Furthermore, in times of disruptive change due to digitalization, people in jobs with a need for human contact know that they are not likely to be replaced by technology in the near future^48^.

Our results on the influencing factors of presenteeism confirm the findings of previous studies from the same^16^ and other sectors showing that quantitative and emotional demands at work as well as the requirement to hide feelings at work and a work environment perceived as stressful (e.g., noise, cold) lead to higher presenteeism in everyday life^33,49–51^. However, we found that healthcare professionals in the German-speaking part of Switzerland report a higher level of presenteeism than their colleagues from the Italian- or French-speaking part. This difference might originate from cultural differences, which were found to be a relevant influencing factor for presenteeism^52,53^. According to Götz, Ebert and Rentfrow^54^, people living in the German-speaking part of Switzerland showed greater conscientiousness than people from the other regions^54^. In particular, it was revealed that people who were more conscientious were absent from work less frequently^55^. However, in our study we do not measure individuals’ work attitudes, whose impact on presenteeism has been expected to be relevant but has been insufficiently described until now^3,34^. The findings suggest that future research should explore the overall effect of the influencing factors rather than their independent role by considering individuals’ work attitudes. This could be of particular relevance since the labor market is becoming increasingly globalized, and companies have to establish occupational health management across countries with differing culture-related work attitudes. In regard to the legitimacy of absenteeism, cross-country differences were found^56^. These differences, therefore, may also apply to presenteeism, since individuals from a country in which absenteeism is seen as least acceptable could show higher levels of presenteeism. Future findings reporting the influencing factors controlled for cultural aspects may better help to identify the factor with the highest potential leverage effect and deliver implications for adapting measures to the respective culture. This may contribute to the development of effective interventions as current interventions neglect psychosocial factors^8^.

### Strengths and limitations

This study compares, for the first time, presenteeism between the settings in the health sector as well as between healthcare professionals, based on a large study sample. It contributes to a more comprehensive understanding of the influencing factors of presenteeism in the health sector. Furthermore, the study highlights the need to incorporate individuals’ work attitude into the empirical advancement of the presenteeism framework^3^.

There are also limitations to be considered. First, the cross-sectional design does not allow causal conclusions to be drawn. In addition, the results (e.g., working hours) are influenced by Swiss labor laws; therefore, results from other countries might differ. Moreover, the study sample is not exactly representative of Switzerland since the German-speaking part is somewhat overrepresented. The study sample matches with the average age and proportion of sex compared to the population of Swiss health professionals. In terms of professional groups, the nurses are overrepresented^46,57^. However, the comparison of the professional groups by setting shows that the descriptives of the study sample correspond to the population. For example, the proportion of nurses in nursing homes or home care is higher than in the other settings. Also, participation was fully voluntary for organizations as well as for healthcare professionals, which probably led to a certain selection bias. Furthermore, presenteeism was measured using a single item, which is mainly the case in research on presenteeism but does not allow for proper psychometric validation^33^. In the case of the single used item, we need to consider possible recall bias due to the long recall period of 12 months^58^. Most of the research conducted measuring presenteeism has a 12-month retrospective focus^8^. However, this may not necessarily qualify as adequate justification for future research to use such a long recall period. The appropriate recall period for presenteeism is still being debated and available scales differ largely between one-week and 12-month recall periods^59^. To reduce the risk of memory loss, the measurement of presenteeism along with its antecedents and consequences is proposed to have daily self-reports or at least be done on a weekly basis^8,60^. Nevertheless, this must be weighed against the associated time commitment for the participants. Further research to compare different recall periods is therefore needed.

Regardless of this ongoing discussion, it seems more appropriate for future measures of presenteeism to try alternative approaches with a more comprehensive underlying construct and the possibility of psychometric validation of the questionnaire^61^. The questionnaire of Hägerbäumer^33^, for example, is less focused on the absolute number of days with presenteeism and more concerned with the extent of presenteeism using examples. However, it is currently only available in German^8^. It also neglects the multidimensionality of presenteeism as it is based on the definition of presenteeism as a behavior of going to work despite being ill.

## Conclusions

Presenteeism differs between disciplines and settings in the health sector. The reasons for presenteeism and its influencing factors in the health sector are mostly consistent with those in other sectors. One aspect that has received little attention so far seems to be cultural differences. Individuals' work attitudes should be included as influencing factors in the future when measuring presenteeism at work. This seems to be particularly relevant for multilingual countries but also elsewhere due to increasing globalization. Alternative approaches to measuring presenteeism should be explored and compared.

## Supplementary Information

Supplementary Information.

## Data Availability

The raw data set analyzed in the current study is available from the corresponding author upon reasonable request.

## References

[1] Miraglia M, Johns G (2016). Going to work ill: A meta-analysis of the correlates of presenteeism and a dual-path model. J. Occup. Health Psychol..

[2] Ospina MB, Dennett L, Waye A, Jacobs P, Thompson AH (2015). A systematic review of measurement properties of instruments assessing presenteeism. Am. J. Manag Care.

[3] Lohaus D, Habermann W (2019). Presenteeism: A review and research directions. Hum. Resour. Manag. Rev..

[4] Collins JJ (2005). The assessment of chronic health conditions on work performance, absence, and total economic impact for employers. J. Occup. Environ. Med..

[5] Evans-Lacko S, Knapp M (2016). Global patterns of workplace productivity for people with depression: Absenteeism and presenteeism costs across eight diverse countries. Soc. Psychiatry Psychiatr. Epidemiol..

[6] Kigozi J, Jowett S, Lewis M, Barton P, Coast J (2017). The estimation and inclusion of presenteeism costs in applied economic evaluation: A systematic review. Value Health.

[7] Igic, I. *et al*. Job-stress index 2014–2016. *Gesundheitsförderung Schweiz* https://friendlyworkspace.ch/system/files/documents/2022-10/Arbeitspapier_043_GFCH_2017-12_-_Job-Stress-Index_2014_bis_2016.pdf (2017).

[8] Ruhle SA (2019). “To work, or not to work, that is the question”—recent trends and avenues for research on presenteeism. Eur. J. Work Organ. Psychol..

[9] Ishimaru T, Mine Y, Fujino Y (2020). Two definitions of presenteeism: Sickness presenteeism and impaired work function. Occup. Med..

[10] Werapitiya, C., Opatha, H. & Fernando, R. Presenteeism: Its importance, conceptual clarifications, and a working definition. In *12th International Conference on Business Management (ICBM)* (2015).

[11] Karanika-Murray M, Biron C (2020). The health-performance framework of presenteeism: Towards understanding an adaptive behaviour. Hum. Relat..

[12] Eurofound. Health and wellbeing at work: A report based on the fifth European working conditions survey’. *Eurofound* https://www.eurofound.europa.eu/publications/report/2013/working-conditions/health-and-well-being-at-work (2012).

[13] Aronsson G, Gustafsson K, Dallner M (2000). Sick but yet at work. An empirical study of sickness presenteeism. J. Epidemiol. Community Health..

[14] Chambers C, Frampton C, Barclay M (2017). Presenteeism in the New Zealand senior medical workforce-a mixed-methods analysis. NZ Med. J..

[15] Boniol, M. *et al*. Gender equity in the health workforce: Analysis of 104 countries. *World Health Organization* https://apps.who.int/iris/handle/10665/311314 (2019).

[16] Allemann A, Siebenhüner K, Hämmig O (2019). Predictors of presenteeism among hospital employees: A cross-sectional questionnaire-based study in Switzerland. J. Occup. Environ. Med..

[17] Freeling M, Rainbow JG, Chamberlain D (2020). Painting a picture of nurse presenteeism: A multi-country integrative review. Int. J. Nurs. Stud..

[18] Homrich PHP, Dantas-Filho FF, Martins LL, Marcon ER (2020). Presenteeism among health care workers: Literature review. Rev. Bras. Med. Trab..

[19] Webster RK (2019). A systematic review of infectious illness presenteeism: Prevalence, reasons and risk factors. BMC Public Health.

[20] Aiken LH (2013). Nurses’ reports of working conditions and hospital quality of care in 12 countries in Europe. Int. J. Nurs. Stud..

[21] Hämmig O (2018). Explaining burnout and the intention to leave the profession among health professionals: A cross-sectional study in a hospital setting in Switzerland. BMC Health Serv. Res..

[22] NIOSH. Exposure to stress: Occupational hazards in hospitals. In *Department of Health and Human Services Centers for Disease Control and Prevention National Institute DHHS (NIOSH)* https://www.cdc.gov/niosh/docs/2008-136/default.html (2008).

[23] Peter KA, Hahn S, Schols JM, Halfens RJ (2020). Work-related stress among health professionals in Swiss acute care and rehabilitation hospitals: A cross-sectional study. J. Clin. Nurs..

[24] Peter KA, Schols JM, Halfens RJ, Hahn S (2020). Investigating work-related stress among health professionals at different hierarchical levels: A cross-sectional study. Nurs. Open.

[25] Eurofound. Work-related stress. *Eurofound* https://www.eurofound.europa.eu/publications/article/2005/work-related-stress (2005).

[26] Kristensen, T. S. A new tool for assessing psychosocial factors at work: The Copenhagen Psychosocial Questionnaire. In *National Institute of Health* https://taskconsult.files.wordpress.com/2016/10/2001-tsk-a-new-tool-for-assessing-psychosocial-factors-at-work-copsoq.pdf (2000).

[27] Kristensen TS, Høgh A, Borg V (2005). The Copenhagen Psychosocial Questionnaire: A tool for the assessment and improvement of the psychosocial work environment. Scand. J. Work Environ. Health.

[28] Nübling, M., Stößel, U., Hasselhorn, H. M., Michaelis, M. & Hofmann, F. Methoden zur erfassung psychischer belastungen: Erprobung eines messinstruments (COPSOQ). In *Bundesanstalt für Arbeitsschutz und Arbeitsmedizin* https://www.baua.de/DE/Angebote/Publikationen/Schriftenreihe/Forschungsberichte/2005/Fb1058.html (2005).

[29] Hasselhorn, H. M., Müller, B. H., Tackenberg, P., Kümmerling, A., & Simon, M. Berufsausstieg bei pflegepersonal: Arbeitsbedingungen und beabsichtigter berufsausstieg bei pflegepersonal in deutschland und Europa. In *Wirtschaftsverlag für Neue Wissenschaften* https://www.baua.de/DE/Angebote/Publikationen/Schriftenreihe/Uebersetzungen/Ue15.html (2005).

[30] Sarason IG, Sarason BR, Shearin EN, Pierce GR (1987). A brief measure of social support—practical and theoretical implications. J. Soc. Pers. Relat..

[31] Sarason IG, Levine HM, Basham RB, Sarason BR (1983). Assessing social support: The social support questionnaire. J. Pers. Soc. Psychol..

[32] Eurofound. Sixth European working conditions survey-questionnaire. *Eurofound* https://www.eurofound.europa.eu/surveys/european-working-conditions-surveys/sixth-european-working-conditions-survey-2015 (2015).

[33] Hägerbäumer, M. *Risikofaktor Präsentismus: Hintergründe und Auswirkungen des Arbeitens Trotz Krankheit* (Springer, 2017).

[34] Johns G (2010). Presenteeism in the workplace: A review and research agenda. J. Organ. Behav..

[35] Mayring, P. *Qualitative Inhaltsanalyse. Grundlagen und Techniken* (Beltz, 2015).

[36] Allison, P. D. *Logistic Regression Using SAS: Theory and Application* (SAS Institute, 2012).

[37] Clark TS, Linzer DA (2015). Should I use fixed or random effects?. PSRM.

[38] Schwarz G (1978). Estimating the dimension of a model. Ann. Statist..

[39] Shekofteh, Y., Panahi, S., Boubaker, O., & Jafari, S. Parameter estimation of chaotic systems using density estimation of strange attractors in the state space. In *Recent advances in chaotic systems and synchronization* (eds. Boubaker, O. & Jafari, S.) 105–124 (Elsevier, 2019).

[40] Nakagawa S, Johnson PCD, Schielzeth H (2017). The coefficient of determination r^2^ and intra-class correlation coefficient from generalized linear mixed-effects models revisited and expanded. J. R. Soc. Interface..

[41] Simon, M. *et al*. Auswertung der ersten befragung der next-studie in deutschland. *Universität Wuppertal* http://www.next.uni-wuppertal.de (2005).

[42] Bundesamt für Statistik. Pflegepersonal: Die schweiz im internationalen vergleich. *Bundesamt für Statistik* https://www.bfs.admin.ch/news/de/2019-0615 (2019).

[43] Elstad JI, Vabo M (2008). Job stress, sickness absence and sickness presenteeism in Nordic elderly care. Scand. J. Public Health..

[44] Shan G, Wang S, Wang W, Guo S, Li Y (2020). Presenteeism in nurses: Prevalence, consequences, and causes from the perspectives of nurses and chief nurses. Front. Psychiatry.

[45] d’Errico A (2013). Low back pain and associated presenteeism among hospital nursing staff. J. Occup. Health..

[46] Merçay, C., Grünig, A. & Dolder, P. Gesundheitspersonal in der schweiz—nationaler versorgungsbericht 2021. bestand, bedarf, angebot und massnahmen zur personalsicherung. *Schweizerisches Gesundheitsobservatorium* https://www.obsan.admin.ch/de/publikationen/2021-gesundheitspersonal-der-schweiz-nationaler-versorgungsbericht-2021 (2021).

[47] WHO. Global strategy on human resources for health: Workforce 2030. *World Health Organization* https://apps.who.int/iris/bitstream/handle/10665/250368/9789241511131-eng.pdf (2016).

[48] Frey CB, Osborne M (2017). The future of employment. How susceptible are jobs to computerisation?. Technol. Forecast. Soc. Change..

[49] Kinman G (2019). Sickness presenteeism at work: Prevalence, costs and management. Br. Med. Bull..

[50] Hansen CD, Andersen JH (2008). Going ill to work: What personal circumstances, attitudes and work-related factors are associated with sickness presenteeism?. Soc. Sci. Med..

[51] Böckerman P, Laukkanen E (2009). What makes you work while you are sick? Evidence from a survey of workers. Eur. J. Public Health.

[52] Garczynski AM, Waldrop JS, Rupprecht EA, Grawitch MJ (2013). Differentiation between work and nonwork self-aspects as a predictor of presenteeism and engagement: Cross-cultural differences. J. Occup. Health Psychol..

[53] Lu L, Cooper CL, Lin HY (2013). A cross-cultural examination of presenteeism and supervisory support. Career Dev. Int..

[54] Götz FM, Ebert T, Rentfrow PJ (2018). Regional cultures and the psychological geography of Switzerland: Person–environment-fit in personality predicts subjective wellbeing. Front. Psychol..

[55] Banks C, Pearson S (2021). Personality, staff attitudes and their association with absenteeism and presenteeism in Australian public sector hospital-based nurses: A cross-sectional study. J. Nurs. Manag..

[56] Addae HM, Johns G, Boies K (2013). The legitimacy of absenteeism from work: A nine-nation exploratory study. Cross Cult. Manag..

[57] Bundesamt für Statistik. Beschäftigung und berufe im gesundheitsbereich. *Bundesamt für Statistik*. https://www.bfs.admin.ch/bfs/de/home/statistiken/gesundheit/gesundheitswesen/beschaeftigung-berufe-gesundheitsbereich.html (2022).

[58] Althubaiti A (2016). Information bias in health research: Definition, pitfalls, and adjustment methods. J. Multidiscip. Healthc..

[59] McGregor A, Caputi P (2022). Presenteeism Behaviour: Current Research, Theory and Future Directions.

[60] Leggett S (2016). Content validity of global measures for at-work productivity in patients with rheumatic diseases: An international qualitative study. Rheumatology.

[61] Brborović H, Brborović O (2017). Patient safety culture shapes presenteeism and absenteeism: A cross-sectional study among Croatian healthcare workers. Arh. Hig. Rad. Toksikol..

