# Supplementary material for: Extent and predictors of presenteeism among healthcare professionals working in Swiss hospitals, nursing homes and home care organizations
Source: Sci Rep. 2023 Jul 25;13:12042. doi: 10.1038/s41598-023-39113-6 (PMC10368704; doi:10.1038/s41598-023-39113-6)
Supplement: Supplementary file 1 — Supplementary Information. [file 41598_2023_39113_MOESM1_ESM.pdf]

Extent and predictors of presenteeism among healthcare professionals working in Swiss hospitals, nursing homes and home care organizations

STROBE Statement—Checklist of items that should be included in reports of ***cross-sectional studies***

	Item No	Recommendation	Information in manuscript	not applicable
**Title and abstract**	1	(*a*) Indicate the study's design with a commonly used term in the title or the abstract	Abstract (Method)	
		(*b*) Provide in the abstract an informative and balanced summary of what was done and what was found	Abstract	
**Introduction**				
Background/rationale	2	Explain the scientific background and rationale for the investigation being reported	Background, paragraph 1-3	
Objectives	3	State specific objectives, including any prespecified hypotheses	Background: paragraph 5, no hypotheses were prespecified	
**Methods**				
Study design	4	Present key elements of study design early in the paper	Method: 'Design'	
Setting	5	Describe the setting, locations, and relevant dates, including periods of recruitment, exposure, follow-up, and data collection	Method: 'Recruitment of healthcare organisations', 'Study sample and 'Data collection'	
Participants	6	(*a*) Give the eligibility criteria, and the sources and methods of selection of participants	Method: 'Recruitment of healthcare organisations', 'Study sample and 'Data collection'	
Variables	7	Clearly define all outcomes, exposures, predictors, potential confounders, and effect modifiers. Give diagnostic criteria, if applicable	Method: 'Questionnaire', 'Data analysis'	
Data sources/ measurement	8*	For each variable of interest, give sources of data and details of methods of assessment (measurement). Describe comparability of assessment methods if there is more than one group	Method: 'Questionnaire',	
Bias	9	Describe any efforts to address potential sources of bias	Method: 'Data analysis', paragraph 3-4, Discussion: 'Strengths and limitations'	

Study size	10	Explain how the study size was arrived at	Method: ‘Recruitment of healthcare organisations’	
Quantitative variables	11	Explain how quantitative variables were handled in the analyses. If applicable, describe which groupings were chosen and why	Method: ‘Data analysis’	
Statistical methods	12	(*a*) Describe all statistical methods, including those used to control for confounding	Method: ‘Data analysis’	
		(*b*) Describe any methods used to examine subgroups and interactions	Method: ‘Data analysis’, paragraph 2	
		(*c*) Explain how missing data were addressed	Method: ‘Data analysis’, paragraph 4	
		(*d*) If applicable, describe analytical methods taking account of sampling strategy		x
		(*e*) Describe any sensitivity analyses		x
**Results**				
Participants	13*	(a) Report numbers of individuals at each stage of study—eg numbers potentially eligible, examined for eligibility, confirmed eligible, included in the study, completing follow-up, and analysed	Results: ‘Sample characteristics’, Table 1	
		(b) Give reasons for non-participation at each stage	Discussion: ‘Strengths and limitations’, paragraph 2	x, voluntary basis, no reasons for non-participation were collected
		(c) Consider use of a flow diagram		x
Descriptive data	14*	(a) Give characteristics of study participants (eg demographic, clinical, social) and information on exposures and potential confounders	Results: ‘Study sample description’, Table 1, information on exposures in Table 2, 3 and 4	
		(b) Indicate number of participants with missing data for each variable of interest	number of participants included in Table 1, no explicit specification of missing values	
Outcome data	15*	Report numbers of outcome events or summary measures	Results: Table 2, 3 and 4	
Main results	16	(*a*) Give unadjusted estimates and, if applicable, confounder-adjusted estimates and their	Method: ‘data analysis’	

		precision (eg, 95% confidence interval). Make clear which confounders were adjusted for and why they were included	Results: Table 4	
		(*b*) Report category boundaries when continuous variables were categorized		x, none continuous variables were categorized
		(*c*) If relevant, consider translating estimates of relative risk into absolute risk for a meaningful time period		x
Other analyses	17	Report other analyses done—eg analyses of subgroups and interactions, and sensitivity analyses	Results: Table 2, 3 and 4	
**Discussion**				
Key results	18	Summarise key results with reference to study objectives	Discussion, paragraph 1	
Limitations	19	Discuss limitations of the study, taking into account sources of potential bias or imprecision. Discuss both direction and magnitude of any potential bias	Discussion: 'Strengths and limitations'	
Interpretation	20	Give a cautious overall interpretation of results considering objectives, limitations, multiplicity of analyses, results from similar studies, and other relevant evidence	Discussion, paragraph 2-5	
Generalisability	21	Discuss the generalisability (external validity) of the study results	Discussion: 'Strengths and limitations', paragraph 2	
**Other information**				
Funding	22	Give the source of funding and the role of the funders for the present study and, if applicable, for the original study on which the present article is based	Declarations: 'Funding'	

*Give information separately for exposed and unexposed groups.

Note: An Explanation and Elaboration article discusses each checklist item and gives methodological background and published examples of transparent reporting. The STROBE checklist is best used in conjunction with this article (freely available on the Web sites of PLoS Medicine at http://www.plosmedicine.org/, Annals of Internal Medicine at http://www.annals.org/, and Epidemiology at http://www.epidem.com/). Information on the STROBE Initiative is available at www.strobe-statement.org.
